# Evaluation of the efficacy of chlorhexidine-alcohol vs. aqueous/alcoholic iodine solutions for the prevention of surgical site infections: a systematic review and meta-analysis

**DOI:** 10.1097/JS9.0000000000002024

**Published:** 2024-08-14

**Authors:** Qiong Yang, Jingxian Sun, Zhao Yang, Sanjay Rastogi, Yan-feng Liu, Bin-bin Zhao

**Affiliations:** a The First Clinical Medical School, Hubei University of Chinese Medicine, Wuhan, China; b Affiliated Hospital of Hubei University of Chinese Medicine, Wuhan, China; c Department of Geriatrics,Hubei Provincial Hospital of Traditional Chinese Medicine, Wuhan, China; d Hubei Provincial Institute of Traditional Chinese Medicine, Wuhan, China; e Hubei Shizhen Laboratory, Wuhan, China; f Department of Proctology, Affiliated Hospital of Shandong University of Traditional Chinese Medicine, Jinan, China; g College of Life Science and Technology, Innovation Center of Molecular Diagnostics, Beijing University of Chemical Technology, Beijing, China; h Boston University Medical Center, Boston, Massachusetts, USA; i Department of Hepatobiliary Surgery, Qilu Hospital of Shandong University, Jinan, China; j Basic Medicine College, Hubei University of Chinese Medicine, Wuhan, China; k Engineering Research Center of TCM Protection Technology and New Product Development for the Elderly Brain Health, Ministry of Education, Hubei University of Chinese Medicine, Wuhan, China

**Keywords:** alcoholic iodine, antiseptic, aqueous iodine, chlorhexidine-alcohol, postoperative wound infection, preoperative skin antiseptics, surgery, surgical site infections

## Abstract

**Background:**

Surgical site infection (SSI) is the prevailing complication that occurs after surgery and significantly escalates healthcare expenses. Published meta-analyses and international standards vary in their recommendations for the most effective preoperative skin antiseptic solution and concentration.

**Objective:**

The aim of this systematic review and meta-analysis is to assess the effectiveness of chlorhexidine-alcohol compared to aqueous/alcoholic iodine solutions in preventing postoperative surgical site infections.

**Methods:**

A systematic search was conducted using four electronic databases (PubMed, Embase, Scopus, and Cochrane Library) to select publications published in peer-reviewed journals. The risk ratio (RR) was calculated, along with their 95% confidence intervals. We assessed heterogeneity using Cochrane *Q* and *I* ^2^ statistics and the appropriate *P* value. The analysis used RevMan 5.4.

**Results:**

The current meta-analysis includes 14 randomized controlled trials (RCTs) comparing either 2–2.5% chlorhexidine-alcohol with aqueous/alcoholic iodine. It was demonstrated that the CAG-using group had an overall lower incidence of postoperative surgical site infections compared to the iodine-using group (RR=0.30, 95% CI=0.20–0.46, *I* ^2^=95%, *P*<0.00001). It exhibits comparable efficacy across various surgical procedures, as evidenced by its RR of 0.25 [95% CI 0.15–0.41], *I* ^2^=51%, and *P*<0.0001 for general surgery, RR=0.47 [95% CI 0.32–0.67], *I* ^2^=82%, *P*=0.0002 for cesarean section and RR of 0.47 [95% CI 0.34–0.65], *I* ^2^=76% and *P*<0.00001 for additional surgical procedures, including neurosurgery, orthopedic surgery, etc.

**Conclusion:**

This meta-analysis suggests using either 2.0–2.5% chlorhexidine in alcohol instead of aqueous, alcoholic iodine to prevent SSIs in adult patients undergoing surgery. Chlorhexidine in alcohol worked effectively for general surgery, cesarean sections, and other surgeries. Thus, preoperative skin cleansing with chlorhexidine-alcohol minimizes postoperative SSIs and bacterial colonization in diverse procedures.

## Introduction

Highlights

- What’s already known about this topic?
  Previous RCTs demonstrate that alcohol-based preoperative skin antiseptics effectively decrease surgical site infections (SSIs).
  Povidone-iodine and chlorhexidine-alcohol have been extensively researched as skin disinfectants due to their broad bactericidal properties.
- What does this study add?
  This meta-analysis recommends the utilization of a solution containing 2.0-2.5% chlorhexidine in alcohol, rather than aqueous or alcoholic iodine, for the purpose of preventing surgical site infections in adult patients having surgery.
  Chlorhexidine in alcohol shown efficacy in general surgery, cesarean sections, and other surgical procedures. It may be inferred that preoperative skin cleansing using chlorhexidine reduces the occurrence of postoperative surgical site infections (SSIs).

Surgical site infection (SSI) is the prevailing postoperative complication and is linked with elevated morbidity, mortality, and healthcare expenses. SSI is the second most prevalent kind of hospital-acquired infection, accounting for ~14–16% of all such infections^1^. An estimated 7–9% of individuals experience post-surgical problems as a result of infection^2^. The infection rate exhibits significant variation based on patient characteristics, including low socioeconomic position, medical conditions, compromised immunity, steroid usage, bleeding, body mass index, operation duration, lack of preventive measures, and emergency surgical procedures^3^. Several external variables contribute to SSI, such as the patient’s skin preparation, hand-cleaning practices, operating room environment, instrument processing, and hospital products utilized in the operating room^4^. Therefore, selecting the appropriate antiseptic for skin preparation is a critical aspect of preventing SSI.

Povidone-iodine and chlorhexidine-alcohol are the most extensively researched skin disinfectants due to their effectiveness against a wide range of microorganisms, including gram-positive bacteria, gram-negative bacteria, viruses, fungi, and *Mycobacterium tuberculosis*. Povidone-iodine is a topical antiseptic that treats and prevents wound infections. Polyvinylpyrrolidone (povidone, PVP) and elemental iodine combine to form this chemically stable compound, with an estimated iodine content ranging from 9.0 to 12.0% based on its dry weight. Nevertheless, povidine iodine can lead to adverse reactions such as localized edema, irritation, pruritus, and dermatitis. Furthermore, excessive use of povidone-iodine, due to its high iodine concentration, may result in corrosive consequences^5^. Similarly, chlorhexidine-alcohol (CHA) is a recently used skin preparation. It typically consists of a combination of either 2–2.5% chlorhexidine gluconate or 70% isopropyl alcohol. Chlorhexidine, on the other hand, may cause adverse effects such as allergic skin reactions, altered taste, increased dental calculus formation, oral discomfort, and swollen glands in the face or neck^6^. The effectiveness of preoperative skin antiseptics in preventing SSIs is widely recognized and is a standard procedure. Nevertheless, there is a current debate regarding the most efficacious antiseptic for the prevention of SSIs. The guidelines from the World Health Organization (WHO)^7^, the UK National Institute for Health and Care Excellence (NICE)^8^, and the US Centers for Disease Control and Prevention (CDC)^9^ have contradictory advice about surgical skin preparation for the prevention of SSIs. The NICE and WHO guidelines advocate for the use of chlorhexidine in alcohol, but the US CDC recommends that any alcohol-based solution can be used. Multiple randomized controlled studies (RCTs) have been carried out, revealing recent evidence regarding this particular subject^10,11^. These RCTs frequently test different types and strengths of skin antiseptics. Various meta-analyses have been performed, including those conducted for the formulation of the NICE and WHO guidelines^12,13^. Both povidone-iodine and chlorhexidine exhibit strong bactericidal activity, but studies suggest that chlorhexidine outperforms in rapidly eliminating surface skin germs, reducing bacterial migration, and exhibiting a faster bactericidal rate. Chlorhexidine is safe and efficient, and comparative research with chlorhexidine, hexachlorophene, and iodophors indicates that chlorhexidine is the most efficacious agent^14^. Previous randomized controlled trials (RCTs) and meta-analyses provide evidence indicating that alcohol-based preoperative skin antiseptics effectively lower the incidence of SSIs. However, these analyses have shown inconsistencies in both the selection of studies and data, and none of the guidelines provide a specific recommendation regarding the appropriate concentration of the recommended antiseptic. Hence, this meta-analysis seeks to offer a proficient and appropriate approach to amalgamate all the existing evidence from 14 different RCTs^15–28^ that compare chlorhexidine in alcohol with aqueous or alcoholic iodine selected as per the prespecified inclusion–exclusion criteria and conducted the simultaneous investigation of these RCTs.

## Objectives

The aim of this systematic review and meta-analysis is to assess the effectiveness of chlorhexidine-alcohol compared to aqueous/alcoholic iodine solutions in preventing postoperative surgical site infections.

## Material and methods

### Search strategy and selection criteria

This systematic review and meta-analysis adhere to the PRISMA (Preferred Reporting Items for Systematic Reviews and Meta-Analyses)^29^ and AMSTAR (Assessing the methodological quality of systematic reviews) Guidelines^30^.

A systematic review of relevant RCTs chosen as per the predefined inclusion and exclusion criteria was conducted to compare the efficacy of two antiseptic skin preparation treatments: chlorhexidine, alcohol, and iodine concentrations in aqueous and alcohol-based solutions. To search the relevant RCTs, a thorough search of the scientific literature databases Embase, PubMed, Scopus, and Cochrane CENTRAL for articles published until 30 March 2024 was conducted. The search terms used were: “surgical site infections”, “SSI”, “pre-operative skin antiseptics”, “chlorhexidine-alcohol”, “CA”, “aqueous”“iodine”, “alcoholic iodine”, “pre-operative care”, “post-operative wound infection”, “antiseptic”, “general surgery”, “caesarean section”, “surgery”, “incidence/rate of surgical site infection”, “adverse events”, “IPA”, “isopropyl alcohol”, “RCT”, “randomized controlled trials”, “wound class”, “systematic review”, and “meta-analysis”.

Following the PICOS criteria^31^, keywords for agreement in both the MEDLINE and Embase databases were identified and assessed. The specified keywords were entered into the Title (ti)-Abstract (abs)-Keyword (keyword) field during the Scopus search. The search terms “surgical site infections,” “pre-operative skin antiseptics,” and “post-operative wound infection” were used in the Cochrane database. The utilization of the PICO framework enabled the creation of exact criteria for selection. The letter “P” was used to designate patients who had undergone surgery. The letter “I” refers to the intervention group that utilized chlorhexidine-alcohol for the purpose of preventing SSI. The letter “C” was used as a control group treated with alcoholic or aqueous iodine. The primary clinical outcomes, denoted by “O,” consisted of the overall incidence rate of surgical site infections (SSI), and any negative events associated with the use of chlorhexidine-alcohol. The research design of the current meta-analysis was restricted exclusively to the use of RCTs.

The methodology employed in our search was derived from the approach utilized in the development of the WHO guideline^32^. Additional articles were identified through the implementation of backward and forward citation monitoring on previously published meta-analyses and included studies. The complete search strategy is outlined in Table 1. The titles and abstracts, as well as the complete texts of potentially qualifying articles, were evaluated separately by two reviewers. The senior author was consulted if necessary, and any discrepancies between the two reviewers were resolved through discussion.

**Table 1:** Database search strategy.

Database	Search strategy
Scopus	#1 “Surgical site infections” OR “SSI” OR “pre-operative skin antiseptics” OR “Chlorhexidine-alcohol” OR “CA” OR “Aqueous Iodine” OR “Alcoholic iodine” OR “Pre-operative care” #2 “Post-operative wound infection” OR “antiseptic” OR “general surgery” OR “Caesarean section” OR “surgery” OR “Incidence/rate of Surgical site infection” OR “adverse events” OR “IPA” OR “Isopropyl alcohol” OR “RCT” OR “randomized controlled trials” OR “ wound class” OR “Systematic review” OR “meta-analysis” #3 #1 AND #2
PubMed	#1 “Surgical site infections” OR “SSI” [MeSH Terms]^**#**^ OR “pre-operative skin antiseptics” [All Fields] OR “Chlorhexidine-alcohol” [MeSH terms] OR “CA” [All fields] OR “Aqueous Iodine” [All Fields] OR “Alcoholic iodine” [All Fields] OR “Pre-operative care” [All fields]. #2 “Post-operative wound infection” [MeSH Terms] OR “antiseptic” [All Fields] OR “general surgery” [All Fields] OR “Caesarean section” [All Fields] OR “surgery” OR “Incidence/rate of Surgical site infection” [All Fields] OR “adverse events” [All Fields] OR “IPA” [All Fields] OR “Isopropyl alcohol” [All Fields] OR “RCT” [All Fields] OR “randomized controlled trials” [All Fields] OR “wound class” OR “systematic review” [All Fields] OR “meta-analysis” [All Fields] #3 #1 AND #2
Embase	#1 “Surgical site infections”/ exp^**$**^ OR “SSI”/ exp OR “pre-operative skin antiseptics”/exp OR “Chlorhexidine-alcohol”/exp OR “CA”/exp OR “Aqueous Iodine”/exp OR “Alcoholic iodine”/exp OR “Pre-operative care”. #2 “Post-operative wound infection”/ exp OR “antiseptic” / exp OR “general surgery”/exp OR “Caesarean section”/exp OR “surgery”/exp OR “Incidence/rate of Surgical site infection”/exp OR “adverse events”/exp OR “IPA”/exp OR “Isopropyl alcohol”/exp OR “RCT”/exp OR “randomized controlled trials”/exp OR “wound class” OR “Systematic review”/exp OR “meta-analysis”/exp #3 #1 AND #2
Cochrane library	#1 (Surgical site infections): ti, ab, kw^**@**^OR (SSI): ti, ab, kw OR (pre-operative skin antiseptics): ti, ab, kw OR (Chlorhexidine-alcohol) ti, ab, kw OR (CA): ti, ab, kw OR (Aqueous Iodine): ti, ab, kw OR (Alcoholic iodine): ti, ab, kw OR (Pre-operative care): ti, ab, kw (Word variations have been searched). #2 (Post-operative wound infection): ti, ab, kw OR (antiseptic): ti, ab, kw OR (general surgery): ti, ab, kw OR (Caesarean section): ti, ab, kw OR (surgery): ti, ab, kw OR (Incidence/rate of Surgical site infection): ti, ab, kw OR (adverse events): ti, ab, kw OR (IPA): ti, ab, kw OR (Isopropyl alcohol): ti, ab, kw OR (RCT): ti, ab, kw OR (randomized controlled trials): ti, ab, kw OR (Systematic review): ti, ab, kw OR (meta-analysis): ti, ab, kw OR (Word variations have been searched) #3 #1 AND #2

# MeSH terms: Medical Subject Headings; $ exp: explosion in Emtree- searching of selected subject terms and related subjects; @ ti, ab, kw: either title or abstract or keyword fields.

### Study selection and data extraction

The current study included RCTs that provided comparative data on the efficacy of 2–2.5% chlorhexidine-alcohol in preventing SSI compared to aqueous or alcoholic iodine solutions. The study did not impose any limitations on the year of publication or the specific language used. However, we specifically accepted studies that satisfied the following inclusion criteria:

1. randomized controlled trial (RCT);
2. patients undergoing surgical procedures in operating rooms;
3. participants must be at least 18 years of age;
4. reported outcomes include the incidence or rate of SSIs, any adverse effects that may arise from the use of chlorhexidine-alcohol or alcoholic/aqueous iodine, and the classification of wounds;
5. include full texts and sufficient data for a 2×2 table.

The following studies were excluded: non-RCTs (case series, case–control study, and cohort study), narrative or expert evaluations, animal studies or trials, trials involving children, and studies that did not provide standard preoperative intravenous antibiotic prophylaxis. Bibliographic references that were outdated, anecdotal, or wholly expert-based were excluded from the evaluation process. Two researchers independently collected the demographic profiles of the patients and event data from the included studies using a predetermined form. The extracted information includes the study author, the year, the country, primary and secondary outcomes, the number of patients in each study group, the type of surgery, the number and type of surgical site infections (SSIs), adverse events, the definition of SSI, and the classification of surgical wounds. We contacted the authors to obtain supplementary information when their data proved insufficient or ambiguous. For instance, elucidation was pursued in the event that the concentration of the antiseptic solution was unknown.

### Risk of bias assessment of included studies

The researchers utilized a standardized questionnaire to assess the studies being analyzed for any possible bias. Two authors independently evaluated the risk of bias in individual studies utilizing the Cochrane risk-of-bias tool, version 2^33^. The tool consisted of five components: randomization-induced bias, bias due to deviations from intended interventions, bias attributed to missing outcome information, bias during outcome evaluation, and bias in selecting the reported outcomes. Two researchers conducted an impartial evaluation to assess potential bias. An additional reviewer took on the responsibility of acting as an arbitrator to resolve any remaining conflicts. Ultimately, the possible bias was assessed and categorized as either “uncertain risk,” “high risk,” or “low risk.” Small-study effects and publication bias were assessed using a comparison-adjusted funnel plot^34^. The statistically significant effect of this bias was confirmed using Begg’s test^35^ conducted by MedCalc software^36^.

### Statistical analysis

The software program Review Manager (RevMan) 5.4^37^ was employed to evaluate and analyze the influence of different continuous and dichotomous results. For each study, risk ratios (RRs), accompanied by 95% confidence intervals (CIs)^38^, were computed in order to assess binary outcomes. The DerSimonian Lair method^39^ was employed to calculate the risk ratio (RR) using a 2×2 table^40^ consisting of event data. Studies that did not report any SSIs in either group were omitted from the quantitative evaluation. Forest plots^41^ were designed to evaluate the influence of different outcome determinants. Statistical methods such as the *I* ^2^ test^42^ and the *χ* ^2^ test^43^, which are accompanied by a *P* value, were used to assess heterogeneity. Since the investigations were conducted under different settings, a random effect model^44^ was used. A *P* value below 0.05 is deemed statistically significant^45^. A HSROC plot^46^ was generated to evaluate the test accuracy of all the included studies. A subgroup analysis was performed to assess the effectiveness of chlorhexidine compared to aqueous iodine, chlorhexidine compared to alcohol-based iodine, chlorhexidine compared to iodine, and aqueous iodine to alcoholic iodine in different types of surgeries.

## Results

### Study selection outcomes

**Figure 1:**
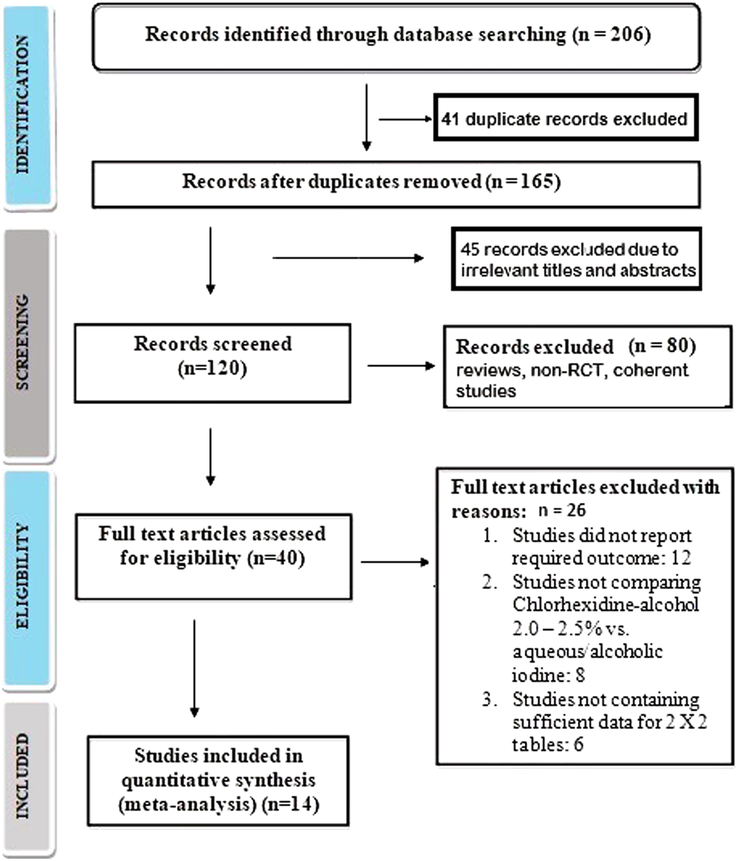
PRISMA study flow diagram.

The search was conducted by performing an exhaustive electronic survey across multiple databases. As a result, 206 studies were identified that met the inclusion criteria outlined in the PICOS paradigm. A total of 165 articles were selected for consideration, while 41 papers were omitted due to duplicate content and inapplicable titles and abstracts. Following further screening, 120 papers were subsequently assessed for eligibility. However, when the inclusion–exclusion criteria were applied, it was found that 80 studies were ineligible and were therefore excluded. The remaining 40 articles were then evaluated to ascertain their eligibility. Out of the total number of studies considered, 26 were excluded on the primary basis of failing to meet the inclusion criteria, lacking sufficient data to generate 2×2 tables, or lacking significant outcome measures. Finally, as shown in Figure 1, 14 RCTS that satisfied the predetermined inclusion–exclusion criteria were ultimately included in this meta-analysis. The included studies comprise a total of 7255 participants who are 18 years of age or older. Nine of the 14 included studies compare the effectiveness of chlorhexidine-alcohol (either 2–2.5%) to aqueous iodine^15,17–20,23,24,27,28^, and the remaining five studies compare the effectiveness of chlorhexidine-alcohol (either 2–2.5%) to alcoholic iodine^16,21,22,25,26^ in preventing the incidence of SSI. The demographic characteristics of the articles included in this meta-analysis are detailed in Table 2. The content presents an account of the author ID and year, study design, comparing antiseptics, total number of participants, age of participants, participants in the chlorhexidine-alcohol using group (CAG) and aqueous or alcoholic iodine-using group (IG), the incidence of SSI, treatments 1 and 2, type of surgery, primary and secondary outcomes and definitions of SSI. Furthermore, event data for the 2×2 table were retrieved from the aforementioned studies in order to perform a meta-analysis.

**Table 2:** Characteristics of the included RCTs.

Author ID and year	Study design	Comparison	Total number of participants	Age of participants	Participants in two groups	SSI/*N* total	Treatment 1	Treatment 2	Type of surgery	Primary and secondary outcomes	SSI definition
Bibi *et al*., 2015^15^	RCT	Chlorhexidine-alcohol 2.0–2.5% vs. aqueous iodine	388	≥18	461 in CAG and 471 in IG	34/388	2.0% CHG in 70% IPA	10% PI (=1% AI)	General surgery	PO: overall rate of SSI SO: Organism growth on the swabs taken (swabs 1, 2, and 3).	CDC
Broach *et al*., 2017^16^	RCT	Chlorhexidine-alcohol 2.0–2.5% vs. alcoholic iodine	802	≥18	392 in CAG and 396 in IG	172/802	2.0% CHG in 70% IPA	0.7% AI in 74.0% IPA	Colorectal surgery	PO: overall rate of SSI SO: Organism growth on the swabs taken (swabs 1, 2, and 3).	CDC
Danasekaran *et al*., 2017^17^	RCT	Chlorhexidine-alcohol 2.0–2.5% vs. aqueous iodine	120	50.02±12.02	60 in CAG and 60 in IG	16/120	2.0% CHG in 70% IPA	5.0% PI (=0.5% AI)	General surgery	PO: overall rate of SSI SO: Organism growth on the swabs taken (swabs 1, 2, and 3).	#C
Darouiche *et al*., 2010^18^	RCT	Chlorhexidine-alcohol 2.0 – 2.5% vs. Aqueous iodine	897	≥18	409 in CAG and 440 in IG	110/897	2.0% CHG in 70% IPA	10% PI (=1% AI)	General surgery	PO: overall rate of SSI SO: Organism growth on the swabs taken (swabs 1, 2, and 3).	CDC
Kunkle *et al*., 2015^19^	RCT	Chlorhexidine-alcohol 2.0–2.5% vs. aqueous iodine	60	≥18	33 in CAG and 27 in IG	3/60	2.0% CHG in 70% IPA	10% PI (=1% AI)	Cesarean section	PO: overall rate of SSI SO: Organism growth on the swabs taken (swabs 1, 2, and 3).	#E
Luwang *et al*., 2020^20^	RCT	Chlorhexidine-alcohol 2.0–2.5% vs. aqueous iodine	311	28.17±4.75	153 in CAG and 158 in IG	21/311	2.0% CHG in 70% IPA	10% PI (=1% AI)	Cesarean surgery	PO: overall rate of SSI SO: Organism growth on the swabs taken (swabs 1, 2, and 3).	#B
Ngai *et al*., 2015^21^	RCT	Chlorhexidine-alcohol 2.0–2.5% vs. alcoholic iodine	1404	≥18	463 in CAG and 474 in IG	60/1404	2.0% CHG in 70% IPA	0.83% AI in 72.5% IPA	Cesarean section	PO: overall rate of SSI SO: Organism growth on the swabs taken (swabs 1, 2, and 3).	CDC
Ritter *et al*., 2020^22^	RCT	Chlorhexidine-alcohol 2.0–2.5% vs. alcoholic iodine	279	≥18	112 in CAG and 167 in IG	26/279	2.0% CHG in 70% IPA	1.0% PI (=0.1% AI) in 70% IPA	Orthopedic surgery	PO: overall rate of SSI SO: Organism growth on the swabs taken (swabs 1, 2, and 3).	#F
Springel *et al*., 2017^23^	RCT	Chlorhexidine-alcohol 2.0–2.5% vs. aqueous iodine	932	≥18	461 in CAG and 471 in IG	62/932	2.0% CHG in 70% IPA	0.75% AI scrub+10% PI paint (=1% AI)	Cesarean section	PO: overall rate of SSI SO: Organism growth on the swabs taken (swabs 1, 2, and 3).	CDC
Sistla *et al*., 2010^24^	RCT	Chlorhexidine-alcohol 2.0–2.5% vs. aqueous iodine	556	≥18	278 in CAG and 278 in IG	33/556	2.5% CHG with 70% ethanol	10% PI (=1% AI)	Inguinal hernia repair	PO: overall rate of SSI SO: Organism growth on the swabs taken (swabs 1, 2, and 3).	CDC
Savage *et al*., 2012^25^	RCT	Chlorhexidine-alcohol 2.0–2.5% vs. alcoholic iodine	100	≥18	50 in CAG and 50 in IG	0/100	2.0% CHG in 70% IPA	0.7% AI in 74.0% IPA	Neurosurgery	PO: overall rate of SSI SO: Organism growth on the swabs taken (swabs 1, 2, and 3).	None
Tuuli *et al*., 2016^26^	RCT	Chlorhexidine-alcohol 2.0–2.5% vs. alcoholic iodine	1147	≥18	572 in CAG and 575 in IG	84/1147	2.0% CHG with 70% IPA	8.3% PI (=0.83% AI) in 72.5% IPA	Cesarean section	PO: overall rate of SSI SO: Organism growth on the swabs taken (swabs 1, 2, and 3).	#G
Xu *et al*., 2017^27^	RCT	Chlorhexidine-alcohol 2.0–2.5% vs. aqueous iodine	159	≥18	79 in CAG and 80 in IG	3/159	2.0% CHG in 70% IPA	10% PI (=1% AI)	Orthopedic surgery	PO: overall rate of SSI SO: Organism growth on the swabs taken (swabs 1, 2, and 3).	#D
Yeung *et al*., 2013^28^	RCT	Chlorhexidine-alcohol 2.0–2.5% vs. aqueous iodine	100	≥18	50 in CAG and 50 in IG	5/100	2.0% CHG in 70% IPA	7.5% iodine scrub+10% PI paint (=1% AI)	Urological implant surgery	PO: overall rate of SSI SO: Organism growth on the swabs taken (swabs 1, 2, and 3).	None

SSI definitions:

CDC: Replicating germs in a wound causes infection at the surgical site, resulting in tissue damage.

#B: purulent discharge from the incision site, wound dehiscence, localized pain or tenderness, localized swelling, and erythema or heat within 30 days following cesarean section.

#C: for example; purulent/serous discharge from the wound, redness of the surrounding area, pain associated with discharge, increased local temperature, within 10 days of surgery.

#D: need for antibiotics or surgical intervention, within 6 weeks of surgery.

#E: presence of purulent drainage, cellulitis, or the need for incision and drainage, or treatment with antibiotics for a clinical diagnosis of infection within 2 weeks of surgery.

#F: wound healing disorders: when CDC criteria were met; SSIs are diagnosed when CDC criteria plus one of the following criteria were met: (1) necessity of antibiotic therapy, (2) necessity of surgical intervention, (3) positive microbiological culture of swabs taken intraoperatively.

#G: the patient reporting the requirement of antibiotic use for a wound infection or documented wound infection in the medical record at the outpatient visit within 30 days of discharge.

CAG, chlorhexidine-alcohol group; CDC, the U.S. Centers for Disease Control and Prevention; I, iodine; IG, iodine group; IPA, isopropyl alcohol; PO, primary outcome; RCT, randomized controlled trial; SO, secondary outcome; SSI, surgical site infection.

### Quality assessment of included studies

**Figure 2:**
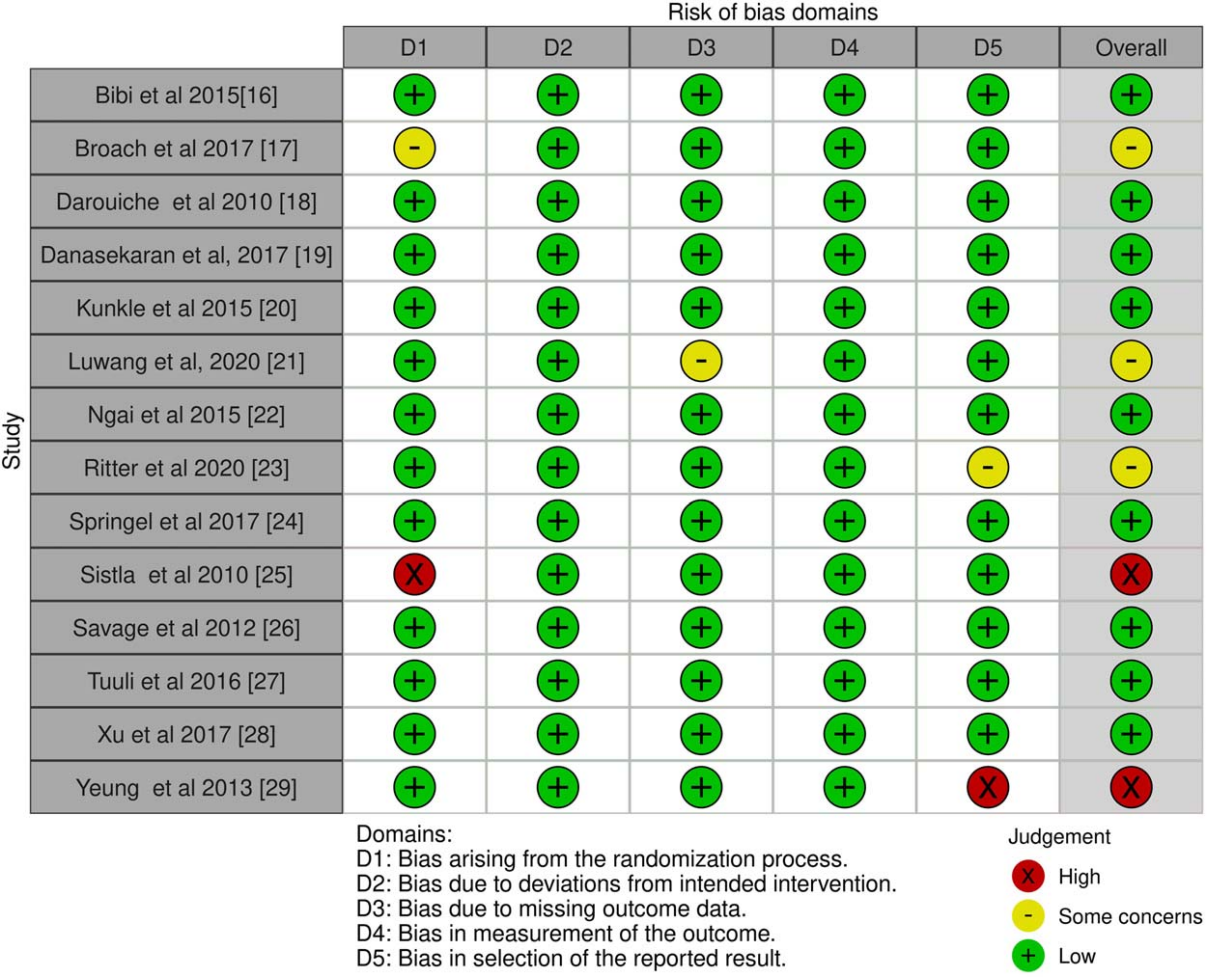
Risk of bias summary plot.

**Figure 3:**
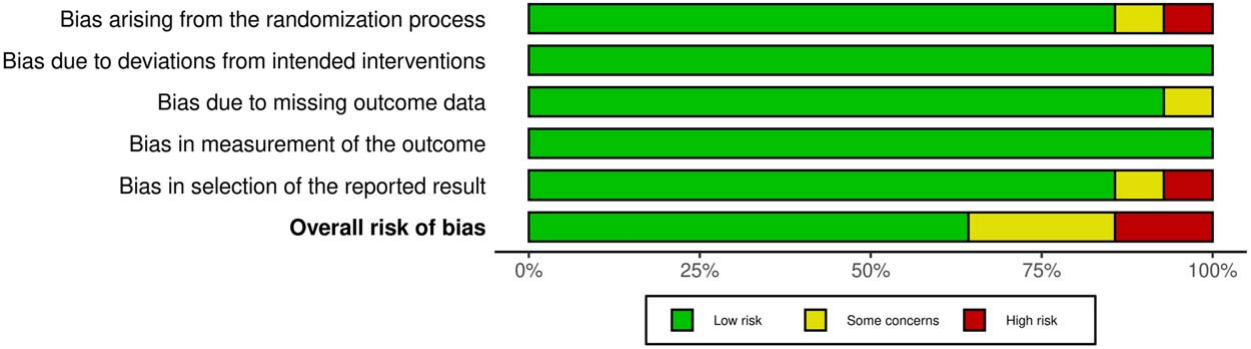
Traffic light plot for assessment of risk of bias.

A risk of bias evaluation was conducted to determine the overall score for the quality of the study. Table 3 presents the outcomes of the risk of bias evaluation for each of the 14 RCTs that were included, utilizing the pre-established questionnaire. The present meta-analysis demonstrates a low risk of bias, as evidenced by the summary plot in Figure 2 and the traffic light plot for bias assessment, as shown in Figure 3. Out of the 14 RCTs, 9 studies were identified as having a low risk of bias. Three RCTs, namely Broach *et al*.^16^, Luwang *et al*.^20^, and Ritter*et al*.^22^, demonstrate a moderate risk of bias. This is attributed to issues with the randomization method, missing outcome data, and the selection of reported outcomes, respectively. The other two RCTs were conducted by Sistla *et al*.^24^ and Yeung *et al*.^28^ demonstrate a high risk of bias pertaining to the randomization method and the selection of reported outcomes, respectively.

**Table 3:** Risk assessment of included studies.

Study ID and year	Bibi *et al*., 2015^15^	Broach *et al*., 2017^16^	Darouiche *et al*., 2010^17^	Danasekaran *et al*., 2017^18^	Kunkle *et al*., 2015^19^	Luwang *et al*., 2020^20^	Ngai *et al*., 2015^21^	Ritter *et al*., 2020^22^	Springel *et al*., 2017^23^	Sistla *et al*., 2010^24^	Savage *et al*., 2012^25^	Tuuli *et al*., 2016^26^	Xu *et al*., 2017^27^	Yeung *et al*., 2013^28^
Was a consecutive or random sample of patients enrolled?	Y	Y	Y	Y	Y	Y	Y	Y	Y	Y	Y	Y	Y	Y
Did the study avoid inappropriate exclusions?	Y	Y	Y	Y	Y	Y	Y	Y	Y	Y	Y	Y	Y	Y
Did all patients receive the same reference standard?	Y	Y	Y	Y	Y	Y	Y	Y	Y	Y	Y	Y	Y	Y
Were all patients included in the analysis?	N	N	N	N	N	N	N	N	N	N	N	N	N	N
Was the sample frame appropriate to address the target population?	Y	Y	Y	Y	Y	Y	Y	Y	Y	Y	Y	Y	Y	Y
Were study participants sampled in an appropriate way?	Y	Y	Y	Y	Y	Y	Y	Y	Y	Y	Y	Y	Y	Y
Were the study subjects and the setting described in detail?	Y	Y	Y	Y	Y	Y	Y	Y	Y	Y	Y	Y	Y	Y
Were valid methods used for the identification of the condition?	Y	Y	Y	Y	Y	Y	Y	Y	Y	Y	Y	Y	Y	Y
Was the condition measured in a standard, reliable way for all participants?	Y	Y	Y	Y	Y	Y	Y	Y	Y	Y	Y	Y	Y	Y

# Y, yes; N, no.

### Primary findings of the included RCTs

Table 4 lists the main outcomes of the included RCTs, including the wound class classification, the incidence of SSIs in the CAG and IG groups with corresponding *P* values, and the adverse events related to antiseptics, such as pruritus, allergy, and erythema.

**Table 4:** Primary results of the included studies.

		Primary results [incidence of SSI (%)]			
Author ID and year	Wound class^a^	CAG (%)	IG (%)	*P*	Adverse events
Bibi *et al*., 2015^15^	1, 2	7.1	10	0.032	Two patients with mild allergic symptoms
Broach *et al*., 2017^16^	2	15.9	18.7	0.030	No events
Danasekaran *et al*., 2017^17^	1, 2	3.33	23.33	0.001	No events
Darouiche *et al*., 2010^18^	2	9.5	16.1	0.004	Three patients with pruritus, erythema, or both around the surgical wound
Kunkle *et al*., 2015^19^	1, 2	11.1	48.5	0.0023	No events
Luwang *et al*., 2020^20^	1, 2	9.5	16.1	0.004	No events
Ngai *et al*., 2015^21^	2	4.6	4.5	0.008	No events
Ritter *et al*., 2020^22^	1	4.5	12.6	0.022	No events
Springel *et al*., 2017^23^	1, 2	6.3	7	0.04	No events
Sistla *et al*., 2010^24^	1	7	9.5	0.04	No events
Savage *et al*., 2012^25^	1	32	34	0.02	No events
Tuuli *et al*., 2016^26^	1,2	4	7.3	0.02	19 patients with erythema at operative site, skin irritation, allergic skin reaction or skin irritation or allergic skin reaction
Xu *et al*., 2017^27^	1	3.8	26.3	0.001	No events
Yeung *et al*., 2013^28^	2	8	32	0.009	No events

^a^ Wound class: 1, clean; 2, clean-contaminated; 3, contaminated; 4, dirty.

#### Wound class classification

The included RCTs: Bibi *et al*.^15^, Danasekaran *et al*.^17^, Kunkle *et al*.^19^, Luwang *et al*.^20^, Springel *et al*.^23^, and Tuuli *et al*.^26^ presented both type 1 (clean) and type 2 (clean and contaminated) classes of wound, while RCTs of Ritter *et al*.^22^, Sistla *et al*.^24^, Savage *et al*.^25^, and Xu *et al*.^27^ reported type 1 (clean) class of wound, and RCTs of Broach *et al*.^16^, Darouiche *et al*.^18^, Ngai *et al*.^21^, and Yeung *et al*.^28^ reported type 2 (clean and contaminated) class of wound. None of the included RCTs present type 3 (contaminated) or type 4 (dirty) wounds.

#### Incidence of SSIs in the CAG and IG groups

All the included RCTs reported that compared to the CAG group, the incidence of SSI was significantly higher in the IG group (*P*<0.05).

#### Adverse events related to antiseptics

Only three RCTs reported the occurrence of adverse events like pruritus, allergy, and erythema related to the use of either chlorhexidine-alcohol or aqueous or alcoholic iodine. Bibi *et al*.^15^ reported two patients with mild allergic symptoms, Darouiche *et al*.^18^ reported three patients with pruritus, erythema, or both around the surgical wound, and Tuuli *et al*.^26^ reported 19 patients with erythema at the operative site, skin irritation, allergic skin reaction, or both as adverse events associated with the use of chlorhexidine-alcohol.

### Findings derived from the statistical investigation

In all, 7255 surgical patients from 14 RCTS were included in the current meta-analysis to evaluate the efficacy of pre-operative antiseptic chlorhexidine-alcohol (either 2–2.5%) vs. aqueous/alcoholic iodine in prevention of postoperative SSIs. Following conclusions were obtained from the statistical analysis of the primary study outcome:

#### Comparing aqueous/alcoholic iodine with 2–2.5% chlorhexidine-alcohol for the prevention of postoperative SSIs

**Figure 4:**
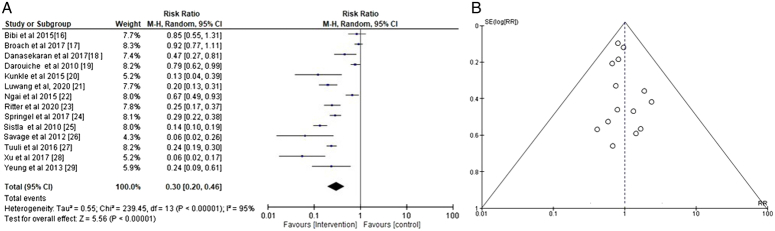
Risk ratio of the overall effectiveness of chlorhexidine-alcohol (2–2.5%) vs. aqueous/alcoholic iodine.

To assess the overall effectiveness of 2–2.5% chlorhexidine-alcohol vs. aqueous/alcoholic iodine in preventing postoperative SSIs, event data from the included studies were used to compute the risk ratio (RR) of SSIs in the CAG and IG groups (Fig. 4). The members of the CAG-using group were shown to have a reduced risk of postoperative surgical site infections than the iodine-using group (Fig. 4A) with a RR of 0.30 [95% CI 0.20–0.46] and *τ* ^2^ value of 0.55, *χ* ^2^=239.45, df=13, *Z*=5.56, *I* ^2^=95%, and *P*<0.00001. Moreover, the symmetrical funnel plots in Figure 4B and a statistically insignificant *P* statistic of Begg’s test (*P*=0.402), which is greater than the predefined significance limit of 0.05, demonstrate a minimal possibility of publication bias.

#### Subgroup analysis

The efficacy of 2.0–2.5% chlorhexidine-alcohol in preventing postoperative wound infections or SSIs was evaluated by subgroup analysis in relation to aqueous iodine, alcohol-based iodine, and various surgical procedures.

**Figure 5:**
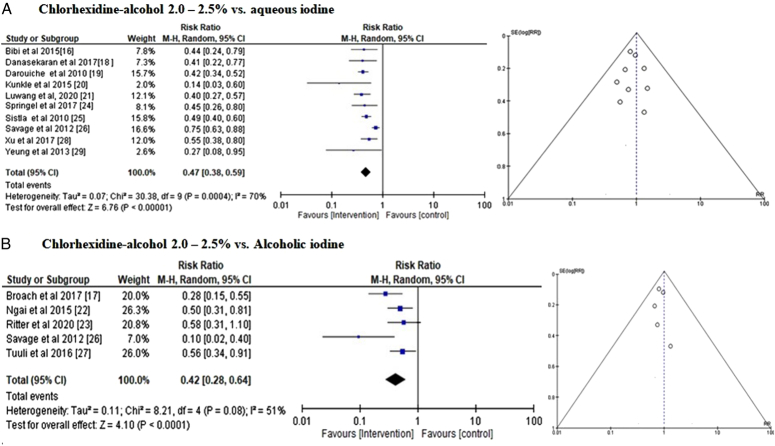
Subgroup analysis risk ratio of (A) chlorhexidine-alcohol (2–2.5%) vs. aqueous iodine. (B) Chlorhexidine-alcohol (2–2.5%) vs. alcoholic iodine.

*Efficacy of 2.0–2.5% chlorhexidine-alcohol vs. aqueous iodine*: To determine how well 2–2.5% chlorhexidine-alcohol vs. aqueous iodine prevents postoperative SSIs overall, event data from the included trials were utilized to calculate the risk ratio (RR) of SSIs in the CAG and IG groups (Fig. 5A). Compared to the iodine-using group, the members of the CAG-using group were revealed to have a lower risk of postoperative surgical site infections with an RR of 0.47 [95% CI 0.38–0.59] and *τ* ^2^ value of 0.07, *χ* ^2^=30.38, df=9, *Z*=6.76, *I* ^2^=70%, and *P*<0.00001. Furthermore, its symmetrical funnel plots and a Begg’s test statistically insignificant *P* statistic (*P*=0.154), which is higher than the predetermined significance criterion of 0.05 show a low likelihood of publication bias.

*Efficacy of 2.0–2.5% Chlorhexidine-alcohol vs. alcoholic iodine*: To evaluate the overall efficiency of 2–2.5% chlorhexidine-alcohol vs. alcoholic iodine in preventing postoperative SSIs, event data from the five included RCTs were utilized to calculate the risk ratio (RR) of SSIs in the CAG and IG groups (Fig. 5B). With an RR of 0.47 [95% CI 0.28–0.64] and *τ* ^2^ value of 0.11, *χ* ^2^=8.21, df=4, *Z*=4.10, *I* ^2^=51%, and *P*<0.00001, the members of the CAG-using group were demonstrated to have a lower risk of postoperative surgical site infections than the iodine-using group. Its symmetrical funnel plots and Begg’s test with a *P* value of 0.231, higher than the pre-established significance standard of 0.05, illustrate a low probability of publication bias.

**Figure 6:**
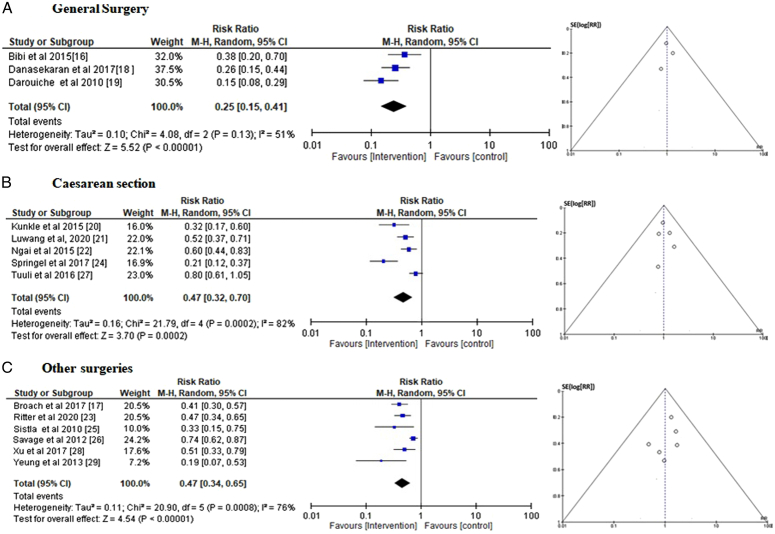
Subgroup analysis of risk ratio of effectiveness of chlorhexidine-alcohol (2–2.5%) vs. aqueous/alcoholic iodine for (A) general surgery, (B) cesarean section, and (C) other surgeries.

*Efficacy of 2.0–2.5% chlorhexidine-alcohol vs. alcoholic iodine for different types of surgery:* Event data from the included trials were used to calculate the risk ratio (RR) of SSIs in the CAG and IG groups for various surgical kinds in order to evaluate the overall efficacy of 2–2.5% chlorhexidine-alcohol vs. aqueous or alcoholic iodine in avoiding postoperative SSIs (Fig. 6). With symmetrical funnel plots, a statistically insignificant *P* statistic of Begg’s test (*P*=0.168), an RR of 0.25 [95% CI 0.15–0.41] and *τ* ^2^ value of 0.10, *χ* ^2^=4.08, df=2, *Z*=5.52, *I* ^2^=51%, *P*<0.0001, the members of the CAG group were shown to have a lower risk of postoperative surgical site infections than the iodine-using group for general surgery (Fig. 6A). With symmetrical funnel plots, a statistically insignificant Begg’s test *P* statistic (*P*=0.233), an RR of 0.47 [95% CI 0.32–0.67] and *τ* ^2^ value of 0.16, *χ* ^2^=21.79, df=4, *Z*=3.70, *I* ^2^=82%, and *P*=0.0002, the members of the CAG group were similarly shown to have a lower risk of postoperative surgical site infections than the iodine-using group for cesarean section (Fig. 6B). With symmetrical funnel plots, a statistically insignificant Begg’s test *P* statistic (*P*=0.189), an RR of 0.47 [95% CI 0.34–0.65] and *τ* ^2^ value of 0.11, *χ* ^2^=20.90, df=5, *Z*=4.54, *I* ^2^=76%, *P*<0.00001 the members of the CAG group were also shown to have a lower risk of postoperative surgical site infections than the iodine-using group for other types of surgeries as well (Fig. 6C).

**Figure 7:**
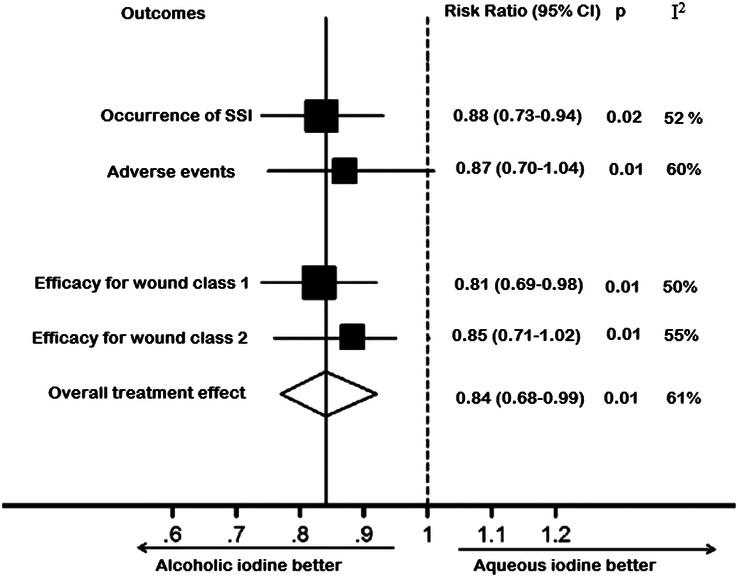
Subgroup analysis of risk ratio of the effectiveness of alcoholic iodine vs. aqueous iodine.

*Efficacy of alcoholic iodine vs. aqueous iodine*: In order to evaluate the efficacy of alcoholic iodine vs. aqueous iodine in the prevention of postoperative SSIs, the risk ratio (RR) of SSIs, adverse events, and efficacy for wound class 1 and wound class 2 in the alcoholic iodine and aqueous iodine groups were calculated (Fig. 7). In comparison to the aqueous iodine-using group, the alcoholic iodine-using group exhibited a lower risk of postoperative surgical site infections (RR=0.88 [95% CI 0.73–0.94; *P*=0.02, *I* ^2^=52%]), low occurrence of adverse events (RR=0.87 [95% CI 0.70–1.04; *P*=0.01, *I* ^2^=60%]), high antiseptic activity for wound class 1 (RR=0.81 [95% CI 0.69–0.98; *P*=0.01, *I* ^2^=50%]). and a wound class 2 RR of 0.85 [95% CI 0.71–1.02; *P*=0.01, *I* ^2^=55%]. The risk ratio value of the aggregate treatment effect is 0.84 [95% CI 0.68–0.99; *P*=0.01, *I* ^2^=61%]. A statistically significant *P* value (*P*<0.05) and an RR-value of less than 1 demonstrate the better effectiveness of alcoholic iodine in the prevention of postoperative SSIs when compared with aqueous iodine.

### HSROC plot for test accuracy of included studies

**Figure 8:**
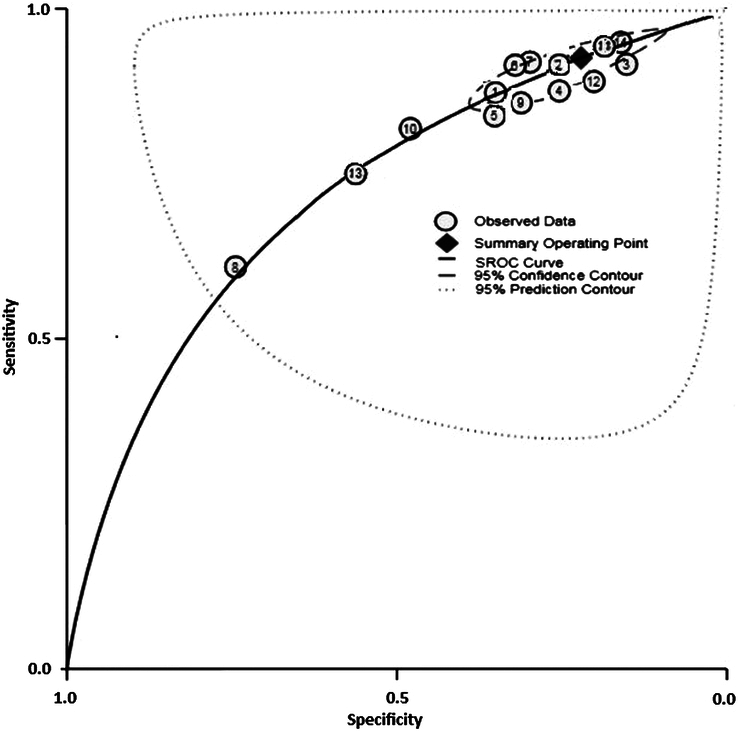
HSROC plot.

The test accuracy of all the studies included was evaluated using the HSROC plot depicted in Figure 8, taking into account the assumption of random effects, which assumes that the unobserved heterogeneity of each individual is not associated with the independent variables. The curve is depicted as a linear line, each examined study is represented by a circular shape, the square represents the point estimate corresponding to the summary sensitivity and specificity, and the dashed line represents the related 95% confidence interval (CI). The regression line represents the curve that summarizes the total diagnostic accuracy. It clearly reveals that the test accuracy of all the included studies is high. The inference can be made based on the clustering of all the data points in the upper left corner, where the sensitivity values are close to one and the specificity values are close to zero. The area under the curve (AUC) of the hierarchical summary receiver operating characteristic (HSROC) was 0.89, with a 95% CI 0.71–0.94 and explains the inherent reliability of diagnostic tests^46^.

## Discussion

Surgical site skin preparation is conducted in the operating room prior to the surgical procedure, immediately preceding the incision and draping to prevent postoperative SSIs. Preoperative skin antiseptic use is a common practice and is well acknowledged to be beneficial in preventing SSIs. Alcohol, chlorhexidine, triclosan, and iodine are antiseptic agents that have the ability to swiftly eliminate both transient and resident microorganisms^47–50^. Evidence suggests that alcohol-based CHG solutions are more effective in reducing SSI rates than alcohol-based povidone-iodine. Chlorhexidine, a cationic bisbiguanide, demonstrates rapid bactericidal activity and maintains its effects by exerting a residual action on the epidermis. In contrast, povidone-iodine is a polymer carrier complex that releases free iodine, which denatures microbial proteins^51,52^. Chlorhexidine in alcohol is indicated by the US CDC (Centers for Disease Control and Prevention) as a potential surgical skin preparation to avoid SSIs^53–55^.

Chlorhexidine works effectively due to its dual modes of action, which include both bacteriostatic (inhibiting bacterial growth) and bactericidal (killing bacteria) effects. Chlorhexidine terminates bacterial cells by disrupting their cell membranes, and the effectiveness of this process is dependent on the concentration of chlorhexidine. Within 30 s of *in-vitro* treatment, chlorhexidine can almost eliminate all gram-positive and gram-negative microbes^56^.

Prior systematic reviews and network meta-analyses have demonstrated that chlorhexidine in alcohol is the most efficacious antiseptic for reducing surgical site infections (SSIs). Wade *et al*.^57^ conducted a systematic review and network meta-analysis that demonstrated that 4% chlorhexidine in alcohol was the most effective antiseptic for reducing SSIs. However, this network meta-analysis was only conducted on clean surgery and misclassified the included studies based on the concentration of chlorhexidine in the alcohol group. While, in their systematic review and meta-analysis, Hasegawa *et al*.^58^ elucidate the potential benefits of an alcohol-based chlorhexidine solution with a concentration of 0.5% or higher for surgical skin preparation in order to prevent SSI. Still, there is ongoing discussion about the best antiseptic to use in preventing SSIs. Therefore, this systematic review and meta-analysis investigated how effectively two distinct skin antiseptic solutions – 2.5% chlorhexidine in alcohol and aqueous or alcoholic iodine solution – lowered the risk of SSI in adult patients undergoing any type of surgery. We hypothesized that a 2–2.5% of chlorhexidine in alcohol would yield the most optimal results in preventing SSIs. To assess the efficacy of 2–2.5% chlorhexidine-alcohol with aqueous or alcoholic iodine solutions in preventing postoperative SSIs, we performed a meta-analysis of 14 RCTs chosen based on our predetermined selection criteria, and compared the risk ratio of postoperative SSI incidence and adverse events in the intervention and control groups. The key findings from the listed research studies are summarized below:

In their RCT, Bibi *et al*.^15^ reported that of the 388 patients from the two institutions, 220 (57%) were in group I and 168 (43%) in group II. In group I, they discovered SSI in 22 patients (10%) and in group II in 12 cases (7.1%) (*P*=0.324). Chlorhexidine was linked with lower infection rates than povidone-iodine, and *Pseudomonas aeruginosa* (23.5%) was the main bacterium linked to SSIs, followed by *Staphylococcus aureus* (17.6%). In their RCT of 788 patients, Broach *et al*.^16^ found that there was a difference in the overall SSI rate between IPA (18.7%) and chlorhexidine-alcohol (15.9%) and that, for clean-contaminated surgery, IPA did not meet the criteria for the effectiveness for overall SSI prevention compared with chlorhexidine-alcohol.

Likewise, in their RCT, Danasekaran *et al*.^17^ reported that initially, there was a *S. aureus* infection (1.67% in the group using CAG and 10% in the group using iodine). Both groups experienced a reduction in bacterial colonization following the application of antiseptic agents, with the chlorhexidine group demonstrating a significant reduction. Darouiche *et al*.^18^ did an RCT with 849 people: 409 were in the chlorhexidine-alcohol group and 440 were in the povidone-iodine group. They found that the overall rate of surgical site infection was 9.5% in the Chlorhexidine-alcohol group compared to 16.1% in the povidone-iodine group (*P*=0.004; relative risk, 0.59; 95% confidence interval, 0.41–0.85). They came to the conclusion that chlorhexidine-alcohol was much more protective than povidone-iodine. Kunkle *et al*.^19^ in their RCT of 60 participants, with 55.0% in the PI group, found that women in the PI group were seven times more likely to have a positive culture (16/33 [48.5%] vs. 3/27 [11.1%] than women in the CG group with an OR of 7.53 [95% CI 1.67–38.83], *P*=0.0023) and reported that the prevalence of positive bacterial cultures obtained at the site of the skin incision 1 was higher in the PI group.

Luwang *et al*.^20^ reported in their study that the rate of SSI in the chlorhexidine-alcohol group was less than 5.4% than that of the povidone-iodine group (8.6%) and *Escherichia coli*, *Klebsiella pneumoniae*, and *Acinetobacter baumannii* were the most common organisms isolated. This study found that the patients who received chlorhexidine-alcohol as a skin antiseptic had a lower chance of developing SSI than those who received povidone-iodine. Ngai *et al*.^21^ in their trial of 1404 women, with 463 in the povidone-iodine group, 474 in chlorhexidine with alcohol (*n*=474), or 467 in both (*n*=467), reported that the overall rate of surgical site infection was less in chlorhexidine with alcohol (4.5%) than in the povidone-iodine group. Ritter *et al*.^22^ reported in their RCT that rates of SSIs were significantly higher in the PVP-I treatment group (12.6%) compared to the CAG treatment group (4.5%) (*P*=0.022). Springel *et al*.^23^ reported in their RCT of 932 subjects (461 assigned to chlorhexidine-alcohol, 471 assigned to povidone-iodine) that surgical site infection occurred in 6.3% of the chlorhexidine-alcohol group and 7.0% in the povidone-iodine group. Ritter *et al*.^22^ reported in their RCT that rates of SSIs were significantly higher in the PVP-I treatment group (12.6%) compared to the CAG treatment group (4.5%) (*P*=0.022).

Springel *et al*.^23^ reported in their RCT of 932 subjects (461 assigned to chlorhexidine-alcohol, 471 assigned to povidone-iodine) that surgical site infection occurred in 6.3% of the chlorhexidine-alcohol group and 7.0% in the povidone-iodine group. Sistla *et al*.^24^ reported in their study that infection rates with the use of the povidone-iodine and chlorhexidine-ethanol groups were not significantly different (9.5 vs. 7.0) and both povidone-iodine and chlorhexidine-ethanol produced a significant reduction in the skin bacterial colony counts. In their RCT comparing chlorhexidine-alcohol 2.0–2.5% with alcoholic iodine in 100 participants over 18 years of age, Savage *et al*.^25^ found that chlorhexidine is an equally effective skin preparation solution for eradication of common bacterial infections. In their RCT of 1147 patients, Tuuli *et al*.^26^ found that surgical site infection was diagnosed in 23 patients (4.0%) in CAG and in 42 (7.3%) in the IG (RR, 0.55; 95% CI 0.34–0.90; *P*=0.02) and determined that the use of chlorhexidine-alcohol for preoperative skin antisepsis resulted in a significantly reduced likelihood of surgical site infection. Concurrently, Xu *et al*.^27^ found that in their RCT comparing Chlorhexidine-alcohol 2.0–2.5% with aqueous iodine in 159 patients over 18 years old, the overall risk of SSI in CAG (3.8%) was lower than 23% in the IG group. Yeung *et al*.^28^ also found in their RCT comparing chlorhexidine-alcohol 2.0 – 2.5% vs. aqueous iodine in 100 patients, less rate of SSI in CAG (8%) as compared to 32% in the IG group. Both concluded that using chlorhexidine-alcohol for preoperative skin antisepsis significantly decreased the risk of surgical site infection.

We conducted a meta-analysis of the event data extracted from the aforementioned RCTs and obtained an RR of 0.30 [95% CI 0.20–0.46], *I* ^2^=95%, *P*<0.00001 for the overall incidence of postoperative SSIs. A risk value less than 1 and a statistically significant *P* value (*P*<0.05) show that the intervention group using 2–2.5% chlorhexidine in alcohol had a lower risk of postoperative SSIs and adverse events after surgery than the control group using aqueous or alcoholic iodine. Furthermore, we also found comparable efficacy of 2–2.5% chlorhexidine in alcohol in reducing the risk of SSIs across various surgical procedures, with RR of 0.25 [95% CI 0.15–0.41], *I* ^2^=51%, and *P*<0.0001 for general surgery, RR=0.47 [95% CI 0.32–0.67], *I* ^2^=82%, *P*=0.0002 for cesarean section, and RR of 0.47 [95% CI 0.34–0.65], *I* ^2^=76%, and *P*<0.00001 for additional surgical procedures, including neurosurgery, orthopedic surgery, etc. Based on these findings, we favor the use of 2–25% chlorhexidine in alcohol as an effective preoperative antiseptic for any kind of surgery in adult patients over aqueous or alcoholic iodine. Our results are consistent with the systematic review, grade assessment, and network meta-analysis of Jalalzadeh *et al*.^59^, who obtained a relative risk of 0·75 (95% CI 0·61–0·92) and concluded that prepping the skin with 2–2.5% chlorhexidine in alcohol is the optimum way to avoid SSIs in adult patients going through surgery, irrespective of the kind of wound they have.

## Limitations

This study emphasizes the implementation of specific search criteria, such as the investigation of “surgical site infections,” “pre-operative skin antiseptics,” and “post-operative wound infection” across many databases. Nevertheless, it is imperative to delineate specific limitations. First of all, it is imperative to recognize the potential selection bias in our analysis as a result of excluding a substantial amount of research. Secondly, the present meta-analysis comprises a mere 14 papers and a limited number of participants included in each subgroup, which exhibit notable heterogeneity and variation. Furthermore, in this investigation, the risk variables for surgical site infections (SSIs) in various procedures, including age, obesity, concurrent illnesses like diabetes and hypertension, and people with immune-compromised conditions, were not considered. Moreover, the influence of preoperative antibiotics and other patient comorbidities was not investigated in the present study. Therefore, it is imperative to carry out further research with a more extensive sample size that considers these risk factors in order to ascertain the benefits of utilizing 2.0–2.5% chlorhexidine in alcohol in comparison to the aqueous or alcoholic iodine for skin antisepsis and the prevention of surgical site infections in adult patients undergoing surgery.

## Conclusion

This meta-analysis presents evidence supporting the use of either 2.0–2.5% chlorhexidine in alcohol over aqueous or alcoholic iodine for preventing surgical site infections in adult patients having surgery. Furthermore, we observed no disparity in the effectiveness of chlorhexidine in alcohol for general surgery, cesarean sections, or other types of surgery. Thus, we can infer that preoperative skin cleansing with chlorhexidine effectively reduces the risk of postoperative SSIs and the colonization of bacteria in various types of surgeries. However, the inclusion of a relatively small number of RCTs limits the current study. Therefore, future research ought to include a greater number of RCTs and larger sample sizes in order to validate these findings and strengthen the overall evidence.

## Ethical approval

Not applicable as the study is totally based on the published literature.

## Consent

Not applicable.

## Source of funding

National Natural Science Foundation of China (82374317), Hubei University of Chinese Medicine Funds for Double First Class Construction (2023ZZXJB004), Shandong Provincial Natural Science Foundation (ZR2021MH339), Hubei Provincial Nature and Science Funding Project (2022CDF143) and Hubei University of Chinese Medicine Funds for Distinguished Young Scholars (2022ZZXJ004).

## Author contribution

Q.Y. and J.S.: concept and designed the study; Z.Y. and Y.L.: analyzed data; B.Z.: collected the data and helped in data analysis; S.R.: drafting of the manuscript, proofreading, and final editing along with guarantor of the manuscript.

## Conflicts of interest disclosure

The authors declare no conflicts of interest.

## Research registration unique identifying number (UIN)

PROSPERO Registration: CRD42024547591.

Review completed, not published.

## Guarantor

Sanjay Rastogi.

## Data availability statement

The datasets used and/or analyzed during the current study are available from the corresponding author on reasonable request.

## Provenance and peer review

Not commissioned, externally peer-reviewed.
